# Ferrimagnetic Clusters as the Origin of Anomalous Curie–Weiss Behavior in ZnFe_2_O_4_ Antiferromagnetic Susceptibility

**DOI:** 10.3390/ma15144789

**Published:** 2022-07-08

**Authors:** Antonio Hernando, Miguel Ángel Cobos, José Antonio Jiménez, Irene Llorente, Asunción García-Escorial, Patricia de la Presa

**Affiliations:** 1 Instituto de Magnetismo Aplicado (UCM-ADIF-CSIC), A6 22,500 Km, 28260 Las Rozas, Spain; antherna@ucm.es (A.H.); micobos@ucm.es (M.Á.C.); 2 Donostia International Physics Center, 20018 Donostia, Spain; 3 IMDEA Nanociencia, 28049 Madrid, Spain; 4 Departamento de Ingeniería, Universidad de Nebrija, 28015 Madrid, Spain; 5 Centro Nacional de Investigaciones Metalúrgicas (CENIM-CSIC), Avda. Gregorio del Amo, 8, 28040 Madrid, Spain; jimenez@cenim.csic.es (J.A.J.); irene@cenim.csic.es (I.L.); age@cenim.csic.es (A.G.-E.); 6 Department of Material Physics, Complutense University of Madrid, 28040 Madrid, Spain

**Keywords:** zinc ferrite, Curie–Weiss temperature, inversion degree

## Abstract

Different studies carried out in the last three decades on the magnetic susceptibility of the spinel ZnFe_2_O_4_ ferrite have revealed the positive character of its Curie–Weiss temperature, contradicting its observed antiferromagnetic behavior which is characterized by a well-defined susceptibility peak centered around the Neel temperature (10 K). Some approaches based on ab initio calculations and mixture of interactions have been attempted to explain this anomaly. This work shows how for very low values of the inversion parameter, the small percentage of Fe atoms located in tetrahedral sites gives rise to the appearance of ferrimagnetic clusters around them. Superparamagnetism of these clusters is the main cause of the anomalous Curie–Weiss behavior. This finding is supported experimentally from the thermal dependence of the inverse susceptibility and its evolution with the degree of inversion.

## 1. Introduction

Many antiferromagnetic materials have been reported to have positive Curie–Weiss temperature, which is a sign of ferromagnetic interactions [1,2,3,4,5,6,7]. This behavior has been explained under the coexistence of competing antiferromagnetic and ferromagnetic interactions. Zinc ferrite (ZnFe_2_O_4_) is a model system for the study of these competing interactions. These spinel ferrites have the formula MFe_2_O_4_, where M is usually one or more divalent or trivalent metals as long as positive charges are compensated for the neutrality of the unit cell. In the general case, the ionic distribution is mixed, and it can be represented by [M_1−δ_Fe_δ_]^A^[M_δ_Fe_2−δ_]^B^O_4_, where δ is the inversion parameter, which specifies the fraction of Fe^+3^ ions in A sites. Accordingly, δ = 0 and 1 stand for the normal and inverse cases, respectively. The normal (Zn)^A^(Fe_2_)^B^O_4_ is antiferromagnetic but the exchange of few Zn-Fe cations between A and B sites gives place to (Zn_1−δ_Fe_δ_)^A^[Zn_δ_Fe_2−δ_]^B^O_4_. Recent evidence of coexisting ferrimagnetic clusters inside of an antiferromagnetic matrix [8,9,10] suggests the use of this material as a model to correlate the increase of Zn–Fe exchange with an anomalous increasing positive Curie–Weiss temperature despite the antiferromagnetic character of the sample.

It is well established that the cation distribution among the interstitial sites of the spinel lattice plays an important role in the magnetic properties of zinc ferrites [8,11,12,13]. These ternary compounds can be described by the formula (Zn_1-δ_Fe_δ_)^A^[Zn_δ_Fe_2-δ_]^B^O_4_,where A and B represent the tetrahedral and octahedral sites, respectively, and δ the inversion parameter. For δ = 0, the structure is a normal spinel and presents a paramagnetic behavior, with a transition to antiferromagnetic order near 10 K. Although the equilibrium cation distribution of bulk zinc ferrites can be assumed to be completely normal, it is very difficult to prevent any exchange between the Zn and Fe cations. Thus, the partially inverted ZnFe_2_O_4_ (δ > 0) has been intensively studied to understand the relationship between cation distribution and magnetic properties [14].

Among the anomalous characteristics of the antiferromagnetic Zn ferrite, the apparent contradiction between the positive Curie–Weiss temperature, determined experimentally from the measurements of the magnetic susceptibility, and the antiferromagnetic behavior [15,16,17] character of both B–B and A–B exchange interactions [9] has been outlined. Different causes have been proposed in an attempt to explain this contradiction [18].

Recent calorimetric and Mossbauer results showed that a very small fraction of Fe atoms occupying tetrahedral A sites can profoundly modify the antiferromagnetic behavior [9]. Specifically, the Mossbauer spectra obtained from samples with a low degree of inversion (δ = 0.05) point to the coexistence of three magnetic structures that can be roughly assigned as: (a) the antiferromagnetic matrix (AFM) with a Neel temperature of the order of 10 K, (b) ferrimagnetic clusters (FM) defined around a tetrahedral or A site occupied by Fe ions, which have a Curie temperature of about 700 K [19], and (c) a spin frustrated or spin disordered (SD) region occupying the boundary between the FM and AFM clusters. It is worth noting that Lotgering [19] took into consideration the existence of ferrimagnetic clusters and calculated the magnetic susceptibility of a single cluster above its Curie temperature by using the molecular field approximation.

In this work, the positive Curie–Weiss temperature obtained from macroscopic measurements of the inverse susceptibility, at temperatures well below the Curie temperature of the ferrimagnetic clusters, is assigned to the superparamagnetic behavior of clusters of atoms with a net magnetic moment originated by A–B superexchange interactions. In other words, the linear thermal dependence of the inverse of susceptibility only appears at temperatures above the blocking temperatures of these superparamagnetic clusters. Thus, the apparent Curie–Weiss temperature is expected to be in the range of the blocking temperatures corresponding to the cluster size distribution. It is important to remark that this temperature is not the critical one corresponding to a phase transition but it corresponds to the temperature at which the relaxation time of the magnetic moment is similar to the measurement time.

## 2. Materials and Methods

As previously reported, the magnetic properties are rather independent of synthesis methods, but strongly depends on the inversion degree [20]. Consequently, different synthesis routes have been carried out to get zinc ferrites with different values of the inversion degree. A samples were obtained from a commercial sample which were annealed at 1100 °C during 24 h and then subjected to different milling times and annealing temperatures, as shown in Table 1. B samples were obtained by the ceramic method mixing ZnO and α-Fe_2_O_3_ in stoichiometric ratio, annealed at 1200 °C for 24 h. Subsequently, the sample was subjected to 50 h of mechanical grinding and annealed for 1 h at different temperatures. C samples were obtained by a mechanical milling of ZnO and α-Fe_2_O_3_ at 1:1 molar ratio for 150 h and subjecting the samples to different annealing temperatures. Table 1 summarizes the synthesis procedures and the subsequent milling times and annealing temperatures and times.

A ball mill Retsch PM4 (Retsch Gmbh, Haan, Germany) was used for the milling of the samples. For more detailed information about the synthesis procedures, see refs. [10,20].

Microstructural characterization of the samples was performed by X-ray diffraction (XRD) measurements at room temperature (RT) using Co radiation (λ = 1.78897Å) in a Bruker (Bruker AXDS, GmbH, Karlsruhe, Germany)AXS D8 Advance diffractometer equipped with a Goebel mirror and a LynxEye linear detector. XRD spectra were collected over an angular range of 2θ from 10 to 120° with a step of 0.01°. The obtained XRD data were refined by the Rietveld method using the version 6.0 of the analysis program TOPAS 6.0 ((Bruker AXDS, GmbH, Karlsruhe, Germany)) and the crystallographic information for zinc ferrite taken from the Pearson crystallographic database [21]. The refinement protocol used included the degree of inversion δ constraining the cations at the octahedral and tetrahedral sites to keep the stoichiometric value. The quality of the refinements was evaluated by the statistically expected least-squares factor (Rexp), the weighted summation of residual of the least-squares fit (Rwp), and the goodness of fit (GoF) or chi-square, whose limit tends to 1. The particle size was determined by SEM and TEM and ranges from a few microns for samples A1 and B1 samples to 15 nm, nanosized particles, NPs, for C2, A3, and B3 samples (see Figure S1 in Supplementary Materials). It must be indicated that TEM observations do not indicate any disturbance at the NPs surfaces that could be associated with a core-shell structure (Figure S3 in Supplementary Materials).

On the other hand, the magnetometric study of the samples was carried out using a standard superconducting quantum interference device (SQUID) MPMS (Quantum Design, GmbH, Darmstadt, Germany). Zero field cooled (ZFC) and field cooled (FC) measurements were taken at magnetic field of 100 Oe between 5 and 300 K, in order to know the behavior as a function of temperature. In addition, hysteresis cycles were measured at 5 and 300 K and at 5 T as maximum applied field.

## 3. Results

**Figure 1:**
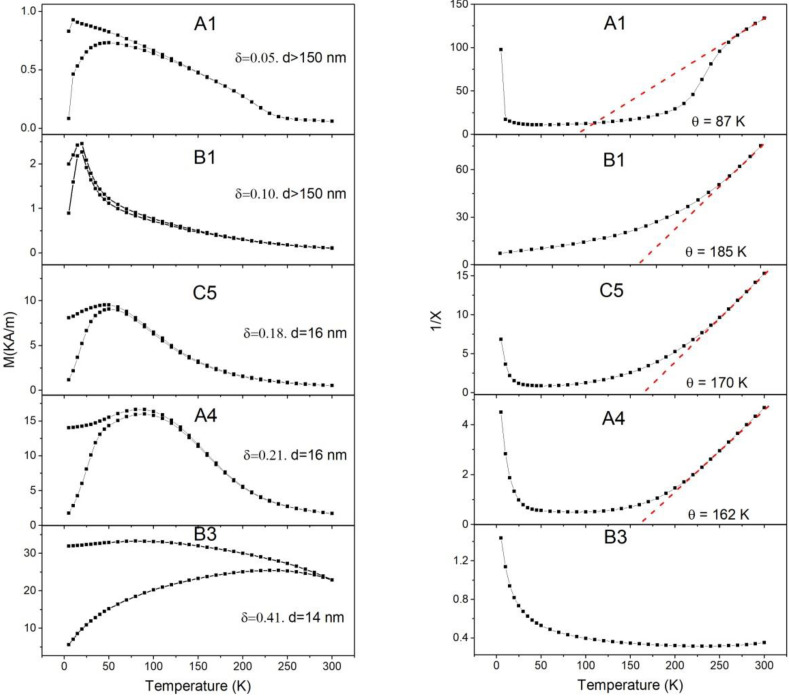
(**Left panel**) shows ZFC-FC curves of zinc ferrites with different inversion degree (δ) and crystallite size (d). The (**right panel**) shows the inverse of the dimensionless susceptibility and the corresponding linear fit with the Curie–Weiss law. The samples labels are identified in Table 2.

Figure 1 shows the temperature dependence of the magnetization under ZFC-FC procedure and the inverse susceptibility curves measured under an applied field of 100 Oe for the samples obtained by different methods with an inversion degree of up to 0.41. As observed, the susceptibility presents a maximum at temperatures that increase with δ. The temperature range corresponding to this maximum is always above the Neel temperature corresponding to the B–B interactions, but well below the Curie temperature corresponding to the A–B coupling. The apparent Curie–Weiss temperature was determined as the intersection with the temperature axis of a tangent line drawn to the inverse susceptibility curve, as shown in the figures on the right panel.

For higher inversion degree values, the maximum of the magnetic susceptibility extends over a wide range of temperatures, so that the inverse of the susceptibility does not present any linear behavior at temperatures below 300 K, as shown in the last couple of graphics in Figure 1 corresponding to a sample (B3) with an inversion degree of around 0.41. Table 2 shows the inversion degree δ, the saturation magnetization at 5 K, apparent Curie–Weiss θ temperatures obtained by fitting the linear behavior of the inverse of the susceptibility, and the blocking temperature calculated from the maximum of the magnetization curves.

## 4. Discussion

It has been reported in previous works that at 2 K, both antiferromagnetic and the ferrimagnetic ordering can coexist for values of the inversion parameter of up to approximately 0.3 [10]. These magnetic orders are associated with AFM coupling between Fe cations, the first among those occupying only octahedral positions and the second among those located in tetrahedral positions with their nearest neighbors in octahedral positions (superexchange B–B and A–B interactions, respectively). As the A–B interaction is almost two orders of magnitude stronger than the B–B one, higher thermal energy is required to disorient their magnetic moments. Thus, magnetic spin clusters may arise in the system from the antiferromagnetic interaction between the Fe cations in the A and B sites above the Neel temperature. At very low inversion degree, most of these clusters are non-contacting to each other and, consequently, are expected to behave as superparamagnetic assemblies or single domains, provided that the cluster volume is smaller than the grain volume. As δ increases, the number of unit cells containing local cation inversions increases, but also the number of inversions of a given cluster could increase while keeping its volume constant. As the blocking temperature only depends on the cluster volume and not on its net magnetic moment, it should increase with δ but not linearly. Accordingly, the experiment with the blocking temperature spreads and the inverse of the susceptibility does not behave linearly with *T* at any range of the measuring interval, as shown in Figure 1 and Table 2 for samples with higher δ values. Therefore, it is assumed that the maximum susceptibility corresponds to the average blocking temperature, <T_B_>, of the ferromagnetic clusters. It is worth noting that T_B_ only depends on the cluster volume and not on the volume of the particles. In fact, the changes of T_B_ with the particle sizes show a tendency opposite to that expected for superparamagnetic particles. This is due to the inverse relation between particle size and average cluster volume. This relation can be understood by taking into account that the smaller the particle size, the larger the inversion degree and, consequently, the larger the average cluster volume, this last being the relevant volume for the superparamagnetic behavior.

As the measuring temperature is well below the Curie point of the ferrimagnetic A–B coupling, i.e., 700 K, the magnetic moment of the clusters can be considered to be constant. When a single Fe atom has jumped from B to A sites in a unit cell, the local δ is increased in an amount of 1/8 [9], and the ferrimagnetic volume of each of these cells is of the order of the unit cell volume, i.e., 6.0 × 10^−28^ m^3^. Therefore, if we consider that there is only one jump per unit cell, the fraction these cells that have undergone an elementary inversion is 40% for a macroscopic inversion value δ = 0.05.

The blocking temperature $T_{B}$ of a ferrimagnetic single domain with volume *V* can be derived from the following, well-known relationship:

(1) $$25k_{B}T_{B}=KV$$

where *K* is the anisotropy constant, $k_{B}$ the Boltzmann constant, *T_B_* the blocking temperature, and *V* the cluster volume. As illustrated by Figure 1, the susceptibility reaches a maximum at temperatures between 10 and 100 K for all the samples. Therefore, if *K* = 1.3 × 10^5^ Jm^−3^ [22] and for instance, it is applied to sample B1 with *T_B_* = 19.9 K, the effective average volume of the ferrimagnetic clusters is in the order of 5 10^−26^ m^3^, which corresponds to a volume of 100 unit cells, that represents single domains of 4 nm size.

Above $T_{B}$, the superparamagnetic susceptibility decreases following the Curie–Weiss law $\chi =C/\left(T-T_{B}\right)$, i.e., in this case *T_B_* = θ.

However, when, as is the case, the volume of the clusters is distributed over a broad range, the inverse of the susceptibility becomes a linear function of T only when T is above the maximum blocking temperature $T_{B}^{\max}$ (Figure S3 in Supplementary Materials). Note that $T_{B}^{\max}$ is that *T* at which all the clusters are superparamagnetic, and it can be also observed and estimated as that for which the ZFC and FC curves become identical. According to this consideration, the experimental susceptibility associated with a cluster distribution with density $n(T_{B})$*,* contains two terms for $T<T_{B}^{\max}$, the statistical average contribution of the unblocked clusters those with $T_{B}<T$, and the contribution of the blocked ones, $\chi_{ferri}$,  which are those with $T_{B}>T$

(2) $$\chi (T)=\frac{1}{3k_{B}}\left[\frac{\mu_{0}n\left(T_{B}\right)m^{2}\left(T_{B}\right)}{T-T_{b}}\right]+n\left(T_{B}\right)\chi_{ferri}\left(T\right)$$

where the average is obtained from $n(T_{B})$*,* distribution that is equivalent to a n(v)*,* distribution according to (1) and <m> is the average magnetic moment of the clusters distribution. In Figure 1, it can be seen that for the sample with inversion degree of 0.41, $T_{B}^{\max}$ is above the measuring *T* interval; in this case, the second term of (2) has an important contribution in all the range of measuring *T* and, consequently, no hints of linear behavior in the inverse of susceptibility versus *T* can be observed.

When the measuring temperature is above $T_{B}^{\max}$, the second term in (2) vanishes, since all the clusters are unblocked, and the inverse of the susceptibility obtained from the first term of (2) asymptotically approaches to linear dependence with *T* as

(3) $$\frac{1}{\chi }=\frac{T-\theta }{C}$$

where θ is close to $T_{B}^{max}$, its exact position depending on the shape and width of the distribution. When the distribution function can be considered uniform $\theta ≅T_{B}^{max}$ (Supplementary Information). The Curie constant, *C*, as well as the macroscopic magnetization *M* can be experimentally obtained, and they are also related by the following expression. Consequently, the average magnetic moment of the cluster distribution can be directly estimated from (4).

(4) $$\left(C=\frac{\mu_{0}nm^{2}}{3k_{B}}=\mu_{0}M\frac{m}{3k_{B}}\right)$$

The spontaneous magnetization *M* is given in Table 2. The experimental *C* values are collected in Table 3 and were inferred by the slope of $\frac{1}{\chi }\;\;$ straight line in Figure 1. It is obvious that as *C* for all the samples is two or three orders of magnitude larger than that corresponding to samples characterized by atomic paramagnetism, showing that it should correspond to canonical superparamagnetism.

The single domain moment depends on the number of uncompensated spins of Fe ions. For a macroscopic δ, the distribution of single domains, according to their volume and magnetic moment, is highly degenerated. However, the order of magnitude of an average *m* value can be inferred by means of expression [4] from the experimental *C* and *M* values.

Expression (4) helps us to infer that *m* should be of the order of 10^2^ µ_B_. Since the maximum magnetic moment per cell corresponding to a local δ = 0.5 becomes 47 µ_B,_ then a group of 2–3 unit cells with δ = 0.5 or a group of 4–6 unit cells with local δ = 0.25 could account for these observations.

It is worth noting that the distance, experimentally found and shown in Table 2, between <T_B_> and θ = $T_{B}^{\max}$, is an index of the width of the clusters size distribution.

Finally, it must be added that the possible presence of magnetostatic interactions among the clusters could contribute to increasing the apparent Curie–Weiss temperature in an amount that, according to the Lorentz field estimation, is expected to be of *C*/3. According to Table 3, the maximum shift produced by the Lorentz field should be of 10 K.

## 5. Conclusions

Due to the antiferromagnetic character of the B–B and A–B superexchange interactions in spinel ferrites, it might be surprising to experimentally find a positive value for the apparent Curie–Weiss temperature. In this work, this behavior has been related to superparamagnetism of magnetic spin clusters nucleated around the Fe^3+^ located in the A sites, which appear in samples with a very low degree of inversion. At temperatures higher than $T_{B}^{max}$, provided that the Curie temperature of the ferrimagnetic clusters is well above the measuring one, the inverse of the susceptibility approaches the typical thermal dependence $\chi =\frac{C}{T-\theta }$. The apparent Curie–Weis temperature, θ, is indeed a temperature corresponding to the blocking temperature distribution of the clusters, its particular position in the spectrum, close to $T_{B}^{max}$, depends on the shape and width of the distribution. In the case of a uniform distribution, θ can be considered to be $T_{B}^{max}$. In summary, in samples with very low inversion degree and blocking temperature well below the Curie one, the contribution of the superparamagnetic effect becomes dominant. The superparamagnetic apparent Curie–Weiss temperature, being a blocking one, is the temperature at which the magnetic relaxation time is similar to the measurement time; above this temperature, the behavior is superparamagnetic but below it, the system is ferromagnetic.

As indicated by Lotgering [19], the measuring temperature should increase up to 800 K to observe the straight line with negative Curie–Weiss temperature due to the antiferromagnetic A–B coupling which gives rise to the ferrimagnetic clusters. On the other hand, the negative Curie–Weiss temperature associated with the B–B antiferromagnetic coupling could only be observed for δ = 0, i.e., for ideal ZnFe_2_O_4_ samples where there is not any trace of ferrimagnetic clusters. However, since this ideal case is very difficult to achieve, the previously reported anomalous sign for the Curie–Weiss temperature can be understood as a consequence of the superparamagnetism associated with the almost unavoidable presence of a few ferrimagnetic clusters.

## Figures and Tables

**Table 1:** Synthesis procedure for the different samples.

Sample Family	Synthesis Route Type	Sample	Thermo-Mechanic Treatment	
			Milling (h)	Annealing (°C)
A	Commercially supplied	A1	-	1100, 24 h
		A2	50	
		A3	50	400, 1 h
		A4	50	500, 1 h
B	Ceramic	B1		1200, 24 h
		B2	2	
		B3	10	
		B4	50	
C	Mechano-Synthesis	C1	150	
		C2	150	300, 1 h
		C3	150	400, 1 h
		C4	150	500, 1 h
		C5	150	600, 1 h

**Table 2:** Inversion degree, saturation magnetization, apparent Curie–Weiss (θ), and average blocking temperature <T_B_> of the studied samples.

Samples	Inversion Parameter	Ms (5 K)	θ	<T_B_>
		(A/m × 10^3^)	(K)	(K)
A1	0.05(2)	12.7(1)	87(5)	14(3)
B1	0.10(2)	80(1)	185(5)	19(3)
C5	0.18(3)	106(1)	170(5)	52(3)
A4	0.21(4)	168(1)	162(5)	88(5)
B2	0.23(5)	164(1)		172(5)
C4	0.26(5)	137(1)		70(5)
A3	0.27(5)	272(2)		129(5) *
B3	0.41(5)	340(3)		235(5) *
C2	0.52(5)	423(4)		270(5) *
C1	0.56(6)	388(4)		>300
B4	0.59(6)	409(4)		263(5)

* Temperatures at which the maximum susceptibility appears but do not allow obtaining a single Blocking Temperature (T_B_).

**Table 3:** Experimental calculation result of m by means slope of 1/X by T ^a^.

Sample	Slope (1/C) Δ(1/X)/ΔT	C	m (µ_B_) (×10^2^)
A1	133.7/213	1.59(2)	5.2(1)
B1	75/115	1.53(2)	0.8(1)
C5	15.3/130	8.5(1)	3.3(1)
A3	4.7/138	29(1)	7.1(2)

^a^ The standard deviations are in parenthesis.

## Data Availability

Not applicable.

## Supplementary Materials

The following supporting information can be downloaded at: https://www.mdpi.com/article/10.3390/ma15144789/s1, Figure S1: At the left, SEM of B1 sample, with d > 150 nm by XRD, and here is appreciated is of micron order (Reprinted with permission from Journal of Alloys and Compounds 849 (2020) 156353. Copyright 2020. Elsevier B.V.). At the right TEM of C1 sample, witch average D size in represented histogram is of 12 nm orders. (Reprinted with permission from The Journal of Physical Chemistry C 2019, 123, (28), 17472-17482. Copyright 2019 American Chemical Society.); Figure S2: HRTEM image of B4 showing that the sample are highly crystalline. (Reprinted with permission from The Journal of Physical Chemistry C 2019, 123, (28), 17472-17482. Copyright 2019 American Chemical Society.); Figure S3: illustrates the thermal dependence of 1/χ as derived from the expression (S2) for TBmin = 30 K, TB^max = 50 K and C = 1. It is shown that θ = TB^max = 50 K. Note that for T sufficiently high, T > T__B_^max, 1/χ, (S2), tends towards a straight line with slope 1/C. As the average blocking temperature is 40K its difference with θ = 50 K is a measure of the half width of the distribution.
