# Supplementary material for: Ferrimagnetic Clusters as the Origin of Anomalous Curie–Weiss Behavior in ZnFe2O4 Antiferromagnetic Susceptibility
Source: Materials (Basel). 2022 Jul 8;15(14):4789. doi: 10.3390/ma15144789 (PMC9317264; doi:10.3390/ma15144789)
Supplement: Supplementary file 1 [file materials-15-04789-s001.zip › materials-1758334-supplementary.pdf]

Supporting Information

Ferrimagnetic Clusters as the Origin of Anomalous Curie–Weiss Behavior in $ZnFe_2O_4$ Antiferromagnetic Susceptibility

Antonio Hernando [1,2,3,4], Miguel Ángel Cobos [1], José Antonio Jiménez [5], Irene Llorente [5], Asunción García-Escorial [5] and Patricia de la Presa [1,6,*]

[1] Instituto de Magnetismo Aplicado (UCM-ADIF-CSIC), A6 22,500 Km, 28260 Las Rozas, Spain; antherna@ucm.es (A.H.); micobos@ucm.es (M.Á.C.)
[2] Donostia International Physics Center, 20018 Donostia, Spain
[3] IMDEA Nanociencia, 28049 Madrid, Spain
[4] Departamento de Ingeniería, Universidad de Nebrija, 28015 Madrid, Spain
[5] Centro Nacional de Investigaciones Metalúrgicas (CENIM-CSIC), Avda. Gregorio del Amo, 8, 28040 Madrid, Spain; jimenez@cenim.csic.es (J.A.J.); irene@cenim.csic.es (I.L.); age@cenim.csic.es (A.G.-E.)
[6] Department of Material Physics, Complutense University of Madrid, 28040 Madrid, Spain
* Correspondence: pmpresa@ucm.es

Structural characterization: SEM and TEM

In the other hand, SEM and TEM analysis were carried out to determine the particle size, see enclosed figure.

In our case the sample particles sizes are in the range of microns for the two samples with very low inversion degree, i.e. A1 and B1, whose crystalline grains is higher than 150 nm. The particle size becomes of the nanometric order of the crystalline grain size for the milled samples with higher inversion degree, i.e. C1 and B4. The relevant sizes for our explanation are the crystalline grain size and the ferrimagnetic cluster size that must be equal or smaller that the grain size in order to be considered as a single domain.

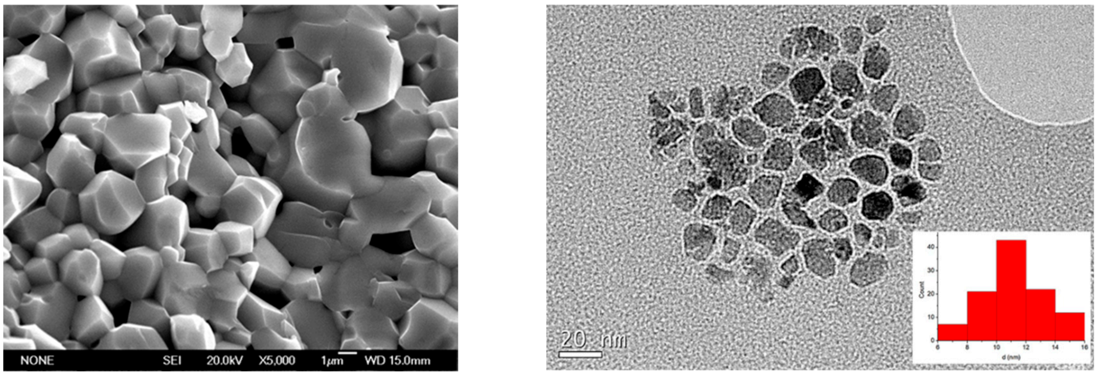

Figure S1. At the left, SEM of B1 sample, with d > 150 nm by XRD, and here is appreciated is of micron order (Reprinted with permission from Journal of Alloys and Compounds 849 (2020) 156353. Copyright 2020. Elsevier B.V.). At the right TEM of C1 sample, witch average D size in represented histogram is of 12 nm orders. (Reprinted with permission from The Journal of Physical Chemistry C 2019, 123, (28), 17472-17482. Copyright 2019 American Chemical Society.)

It is also to be taken into account that electron microscopy observation does not indicate any shell-like disturbance of the crystalline order at the NPs surface, as shown by the enclosed figure by HRTEM.

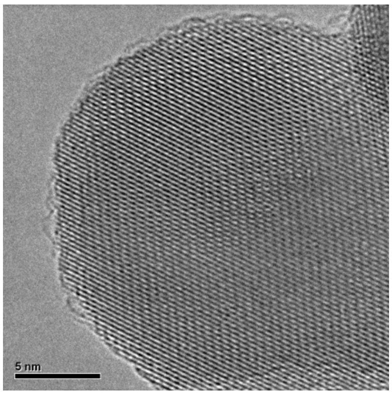

Figure S2. HRTEM image of B4 showing that the sample are highly crystalline. (Reprinted with permission from The Journal of Physical Chemistry C 2019, 123, (28), 17472-17482. Copyright 2019 American Chemical Society.)

Magnetic susceptibility

Let us consider, for the sake of simplicity, a uniform distribution of blocking temperatures in the range between T_B^{min} and T_B^{max} with width $\Lambda = T_B^{max} - T_B^{min}$. The number of clusters per unit volume with blocking temperature comprised between T_B and T_B+dT_B is given by $n(T_B)\, dT_B = (N/\Lambda)\, dT_B$. **N** being the total number of ferrimagnetic clusters per unit volume.

If the measurement of the susceptibility is carried out at a T higher thanT_B^{max}, all the clusters behaves as superparamagnetic and the macroscopic susceptibility turns out to be the sum of all the contributions

$$\chi = \frac{C}{\Lambda}\int_{T_B^{min}}^{T_B^{max}} \frac{dT_B}{T - T_B} \qquad \text{(S1)}$$

It has been assumed that C does not depend on T_B. In fact, T_B is determined by the volume of the cluster that should be correlated to its magnetic moment. However, such correlation is strongly degenerated; for instance, some small clusters can have higher magnetic moments than more big clusters. Therefore, it can be considered that C represents its average value in the assembly of the N clusters.

According to [1´] the inverse of the susceptibility could be approximated to the following expression

$$\frac{1}{\chi} = \frac{\Lambda}{CLn\dfrac{T - T_B^{min}}{T - T_B^{max}}} \qquad \text{(S2)}$$

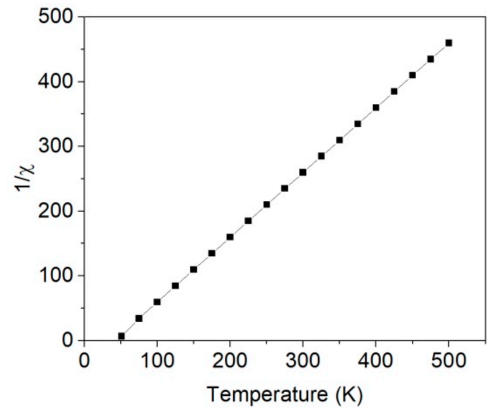

Figure S3. illustrates the thermal dependence of $\frac{1}{\chi}$ as derived from the expression (S2) for T_B^{min} = 30K, T_B^{max} = 50K and C = 1. It is shown that θ=T_B^{max}=50K. Note that for T sufficiently high, T> T_B^{max}, $\frac{1}{\chi}$, (S2), tends towards a straight line with slope 1/C. As the average blocking temperature is 40K its difference with θ = 50K is a measure of the half width of the distribution.
